# Consistency and identifiability of football teams: a network science perspective

**DOI:** 10.1038/s41598-020-76835-3

**Published:** 2020-11-12

**Authors:** D. Garrido, D. R. Antequera, J. Busquets, R. López del Campo, R. Resta Serra, S. Jos Vielcazat, J. M. Buldú

**Affiliations:** 1 Complex Systems Group & GISC, Universidad Rey Juan Carlos, 28933 Madrid, Spain; 2 Laboratory of Biological Networks, Center for Biomedical Technology, UPM, Pozuelo de Alarcón, 28223 Madrid Spain; 3 E.S.A.D.E. Business School, Barcelona, Spain; 4 Mediacoach-LaLiga, 28043 Madrid, Spain; 5 Unmanned Systems Research Institute, Northwestern Polytechnical University, Xi’an, 710072 China

**Keywords:** Complex networks, Nonlinear phenomena

## Abstract

We investigated the ability of football teams to develop a particular playing style by looking at their passing patterns. Using the information contained in the pass sequences during matches, we constructed the pitch passing networks of teams, whose nodes are the divisions of the pitch for a given spatial scale and links account for the number of passes from region to region. We translated football passings networks into their corresponding adjacency matrices. We calculated the correlations between matrices of the same team to quantify how consistent the passing patterns of a given team are. Next, we quantified the differences with other teams’ matrices and obtained an identifiability parameter that indicates how unique are the passing patterns of a given team. Consistency and identifiability rankings were calculated during a whole season, allowing to detect those teams of a league whose passing patterns are different from the rest. Furthermore, we found differences between teams playing at home or away. Finally, we used the identifiability parameter to investigate what teams imposed their passing patterns over the rivals during a given match.

## Introduction

Is it possible to quantify to what extent a team has a defined playing style? During the last years, this question has captured the attention of many scientists working on sports sciences^1–4^. Year after year new technologies are incorporated to obtain more information about any action carried out by players on the pitch. Consequently, the acquisition of event and tracking data has boosted in-depth analysis of football, leading to the definition of new metrics to characterize the player and team performance^5^. Understanding the team’s organization and, particularly, the identification of playing styles has benefited from these new methodologies^6^. For example, departing from the position of all players of a given team during a match, Moura et al.^7^ calculated the convex hull, i.e., the area covered by the contour of the player’s position. Next, they analyzed the convex hull′s fluctuations, quantifying players’ ability to spread and compact depending on the phases of the game, which was linked to a team’s playing style.

Performance indicators have also been successfully used as a proxy to determine playing styles in football. In^8^, several passing parameters (e.g., passes per action, passing directions, and target players) and rates of passing success were combined to define an Index of Game Control (IGC). An Index of Offensive Behaviour (IOB) was also introduced by combining parameters of ball possession, gain of possession and quality of possession. IOB allowed to distinguish between two playing styles, namely, possession play and direct play. Interestingly, IOB revealed that most successful teams preferred possession play. More recently, Fernandez-Navarro et al.^9^ collected 19 team performance indicators (14 during the attacking phase and 5 for the defending one) and automatically classified the playing styles of LaLiga and Premier League teams. Six different factors (e.g., direct/possession play, pressure area, possession width, etc..) were used to define the different playing styles, and the results of the analysis identified 12 different styles according to the score obtained at each factor.

However, despite the advances for an “algorithmic recognition” of the playing style, a fundamental obstacle remains: The team’s playing style is a subjective concept that relates to the team’s overall use of the different playing methods. The team formation, consisting on how players of a team are distributed on the field, has been traditionally used as a proxy to identify the way a team plays. With this regard, the starting line-ups given by the coaching teams to the media at the beginning of a football match are used to describe how players will be organized at the pitch. Classical team formations, such as 4-4-2 or 4-3-3, are then suggested by experienced journalists to be expected during the match. Team formation indicates the position of all players, except for the goalkeeper, towards the opponent’s goal in a qualitative way. For example, the 4-4-2 formation is characterized by 4 defenders (two center-backs in the middle, two full-backs on the left and right sides), 4 midfielders (two central midfielders, two wingers on the left and right sides) and 2 strikers. Teams (and coaches) are supposed to be identified with one (or two) specific team formation, being one of the leading indicators of a team’s playing style. However, nothing is simple in football. As explained by Wu et al.^3^ the formation of players evolves during the match, which recommends the use of tracking algorithms that continuously evaluate the team formation during the different phases of the game.

In this paper, we propose an alternative way of analyzing the playing styles of football teams. Our main aim is to determine whether specific teams maintain their playing style along a season and, more importantly, if it is possible to distinguish the particular organization of a given team from the rest. We focused on questions such as what teams adapt to others and what teams remain loyal to their styles? Or, can we quantify what team imposed its style over its opponents during a given match? To answer these questions, we propose a new methodology to analyze the passing patterns of a given team and evaluate whether they are maintained along a season. Using event data supplied by Mediacoach^10^, we constructed the passing networks associated with each team and analyzed their structures using different methodological tools coming from Network Science^11^.

**Figure 1:**
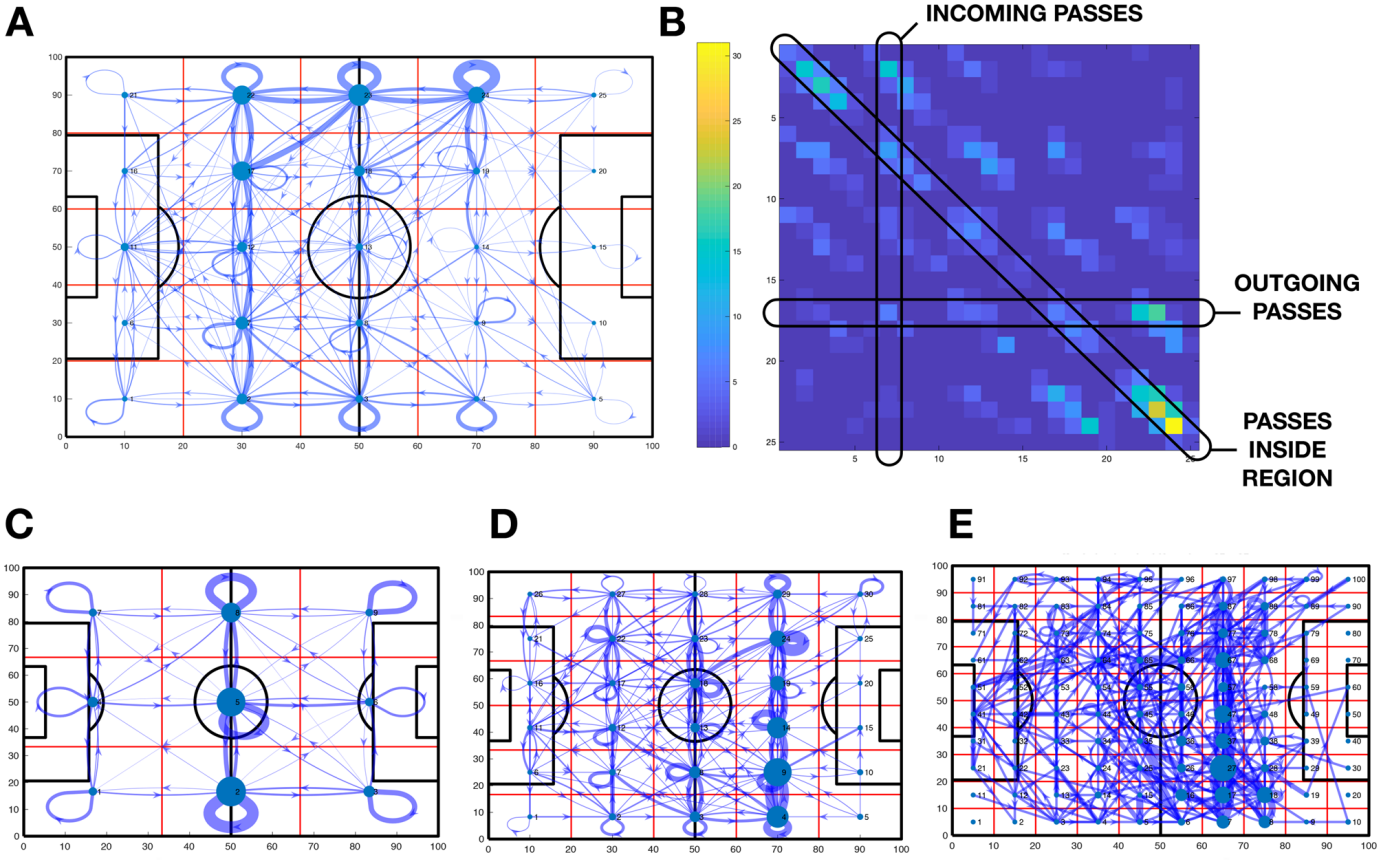
Pitch passing networks. In (**A**), an example of the pitch passing network of Real Madrid during its match against Getafe (season 2018/2019). The pitch is divided into a $p \times q$ grid, leading to $m= p \times q$ nodes. In this particular example, $p=q=5$ and $m=25$. The width of each link is proportional to the number of passes $a_{ij}$ from node (region) *i* to node *j*. In (**B**), we plot the corresponding weighted adjacency matrix $\mathbf{A}$ of the pitch passing network plot in plot (**A**). The element $\mathbf{A} \{i,j\}$ contains the number of passes from node *i* to node *j*. Note that line *k* of the matrix contains all outgoing passes from node *k*. On the other hand, column *k* accounts for the passes entering region *k*. The diagonal elements contain the number of passes starting and finishing inside each of the *k* nodes. In (**C**–**E**), pitch passing networks of Real Madrid during a match against F.C. Barcelona at different scales. Note that, despite passes are the same, the fact that the pitch is divided into areas of different sizes results on different pitch passing networks.

The application of Network Science to a diversity of fields, from epidemics to social networks or brain dynamics, has demonstrated the utility of this new branch of science in analyzing complex problems from new perspectives. In recent years, different research papers have proposed the adaptation of a diversity of network metrics to football^12–14^, showing the possibility of evaluating the performance of players and teams^15–17^. The fundamental difference between Network Science and classical analysis about team organization and performance is that the attention is not paid to the individual components (i.e., players) but, on the contrary, considering the system as a whole. Following this approach, we used the event datasets to construct the *multi-scale pitch passing networks* of football teams. Specifically, we divided the pitch into *m* regions and constructed the pitch-passing networks, whose nodes are the *m* divisions of the pitch and the links between nodes account for the number of passes from one region to any other during a match (see Fig. 1A, for an example). Pitch passing networks have been used to quantify team performance^16^ and analyze differences in the playing patterns of football teams^18^. In our case, we tracked the evolution of the pitch-passing networks of Spanish football teams playing at the first division of *LaLiga* during the season 2018/2019. We defined a *consistency* parameter of each team, which measures how similar the organization of pitch passing networks is during the season. Next, we analyzed how different pitch passing networks are from the rest of the teams, and we defined a new parameter called the team *identifiability*. We borrowed this idea from neuroscience, where identifiability consists of the ability to identify the functional brain networks of one individual from a group of functional networks of different people^19,20^. We propose to use the identifiability parameter to determine what teams have particular passing patterns and what teams change their passing distribution according to their rivals. Finally, we showed that the identifiability parameter could quantify to what extent a team imposed their passing patterns over the other in a given match.

## Methods

### Datasets

Datasets were acquired through Mediacoach^10^, whose event provider is StatsPerform^21^. From all events recorded during a match, we focused in all passes made during each match of the 2018/2019 season of the first division of the Spanish national league *“LaLiga”*. Twenty teams played the competition (see Table 1 for the final ranking). For each of the 380 matches played during the season, we considered the number of successful passes of each team. The information about the passes contained, for a pass *i*: (1) the precise moment *t*(*i*) when the pass was made, (2) the *x* and *y* coordinates of the position from where the pass was sent $[x_1(i)$,$y_1(i)]$ and (3) the position where the pass was received $[x_2(i)$,$y_2(i)]$. Importantly, the *x* and *y* coordinates were given in “field units”, ranging, in both axes, from 0 to 100 (in all fields). The information about the home and away teams of each match was also used in the analysis.

**Table 1:** Teams and ranking at the end of the season 2018/2019 of LaLiga Santander.

Ranking (2018/2019)	Team	Points	Scored goals	Conceded goals
1	F.C. Barcelona	87	90	36
2	Atlético de Madrid	76	55	29
3	Real Madrid	68	63	46
4	Valencia	61	51	35
5	Getafe	59	48	35
6	Sevilla	59	62	47
7	R.C.D. Espanyol	53	48	50
8	Ath. Bilbao	53	41	45
9	Real Sociedad	50	45	46
10	Real Betis	50	44	52
11	Alavés	50	39	50
12	Eibar	47	46	50
13	Leganés	45	37	43
14	Villarreal	44	49	52
15	Levante	44	59	66
16	Valladolid	41	32	51
17	Celta de Vigo	41	53	62
18	Girona	37	37	53
19	S.D. Huesca	33	43	65
20	Rayo Vallecano	32	41	70

Ranking, points, number of scored goals and conceded goals.

### Multi-scale pitch passing networks

Pitch passing networks describe how the ball flows through the field during the possession of a given team using specific partitions of the pitch^18^. The construction of this kind of networks requires several steps, beginning with the division of the pitch into $m= p \times q$ areas, each one corresponding to a node of the passing network, as shown in Fig. 1A. In this particular example, the field was divided into $5 \times 5$ rectangular areas (i.e., $m=25$ areas or nodes). Next, we considered all completed passes made by a given team during a match. The links connecting the nodes of the network represented the number of passes made from each region (i.e., node) to any other. In Fig. 1A arrows are the representation of those links, whose width is proportional to the number of passes between nodes. In turn, we can see in Fig. 1A that some areas have loops, which correspond to passes that start and finish inside the same area. On the other hand, nodes’ sizes are proportional to their importance, which are obtained through the eigenvector centrality, a measure to quantify the relevance of a node inside a network^22^. The adjacency matrix $\mathbf{A}$ is the mathematical abstraction of the pitch passing network, whose elements $a_{i,j}$ account for the number of passes starting in area *i* and finishing in area *j*. The $a_{i,i}$ elements of the diagonal are the number of passes that do not cross the boundaries of the region *i*. Importantly, the adjacency matrix $\mathbf{A}$ has crucial information about each area of the pitch. As we can see in the example of Fig. 1B, the *i*-row of the matrix contains the number of out-going passes of area *i*, while the *i*-column has the number of the incoming passes.

It is worth noting that the number of nodes and, in turn, the structure of the pitch passing networks crucially depends on the number of divisions of the field. In Fig. 1C–E we plot the pitch network of the same team, for the same match, at three different partitions: $3 \times 3$ (C), $5 \times 5$ (D) and $10 \times 10$ (E). Increasing the number of divisions results in an increase of nodes, while the number of passes remains the same. Therefore, the average weight of the links decreases as it does the link density. Indeed, some nodes could get disconnected from the rest when the pitch division leads to a high number of nodes, as shown in the case of Fig. 1E (see the disconnected area at the bottom left). However, we should not consider this fact as a drawback. On the contrary, it means that each scale leads to different networks and, consequently, different information, suggesting a multi-scale analysis of pitch passing networks.

### Calculating consistency and identifiability

The next step was to evaluate if teams’ pitch networks maintain their organization along a season and if networks of certain teams have unique properties not shared with other teams. With this aim, we first analyzed the pitch networks of each team for each match of the season. Specifically, for a given team and given match we obtained the $p \times q$ pitch networks with $1 \le p \le 20$ and $2 \le q \le 20$, leading to passing networks from $m=2$ nodes to $m=400$ nodes. In this way, each pitch network corresponds to a different spatial scale; the higher the number of nodes, the smaller the area associated with them, and the higher the spatial resolution. Next, we computed, for each scale, the correlation between all the adjacency matrices of each team. Given a specific scale *m*, the correlation between two matrices $\mathbf{A}(m)$ and $\mathbf{B}(m)$ is obtained as:

1 $$\begin{aligned} C(m)=\frac{\sum _i \sum _j (\mathbf{A}(m)_{i,j} - \bar{\mathbf{A}}(m)) (\mathbf{B}(m)_{i,j} - \bar{\mathbf{B}}(m))}{\sqrt{\left( \sum _i\sum _j (\mathbf{A}(m)_{i,j} - \bar{\mathbf{A}}(m)) ^2\right) \left( \sum _i\sum _j (\mathbf{B}(m)_{i,j} - \bar{\mathbf{B}}(m)) ^2\right) }} \end{aligned}$$

**Figure 2:**
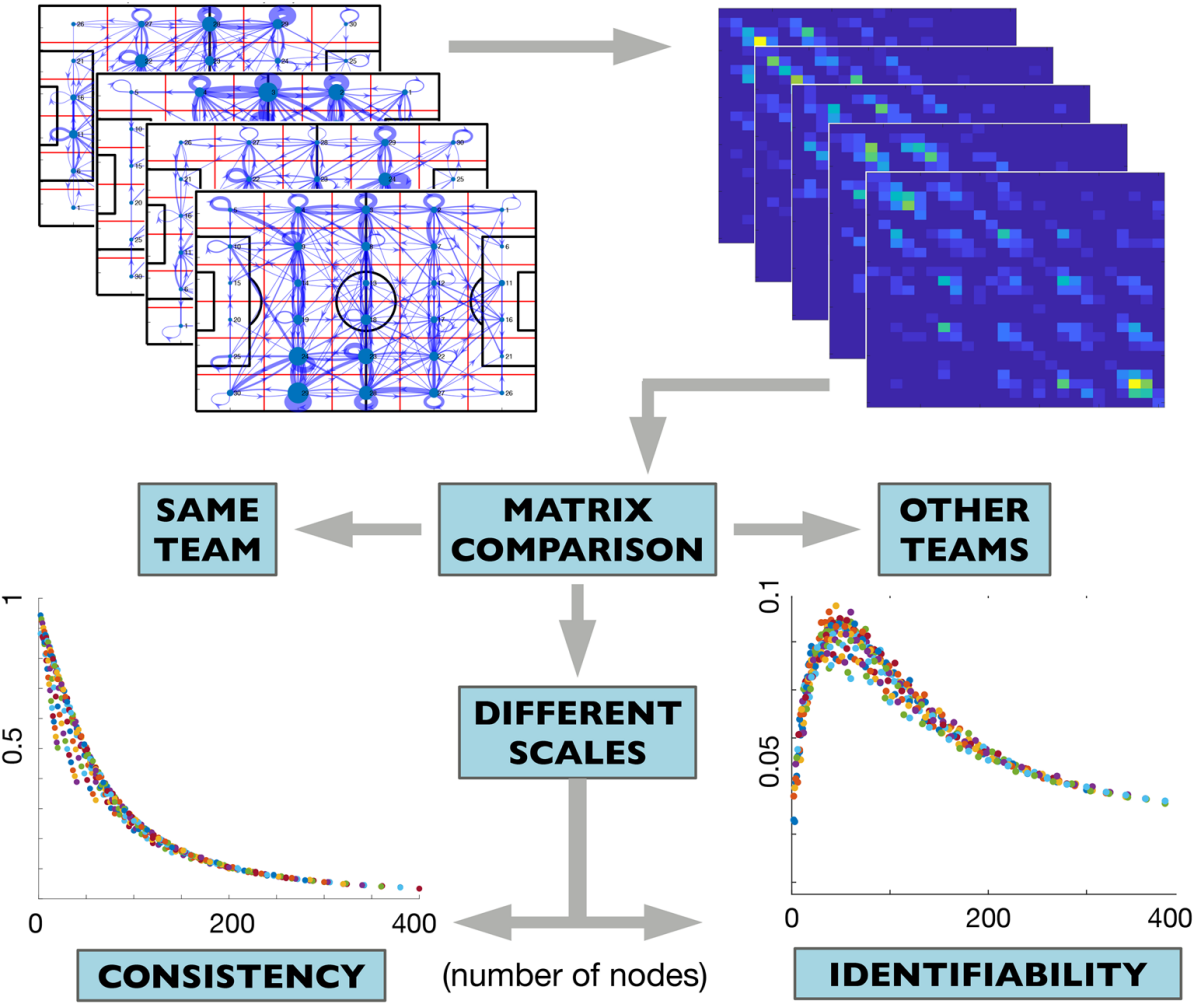
Qualitative description of how the consistency and identifiability of football teams are calculated. First, we obtained the multi-scale pitch passing networks of all matches and teams. Second, the adjacency matrix (see Fig. 1B) of each match and pitch partition is calculated. Third, we calculate the cross-correlation of all matrices for all scales. Each scale corresponds to a different number of nodes *m*. The scale-consistency *C*(*m*) (bottom left) is obtained when only the same team’s adjacency matrices are considered. Finally, we calculate the scale-identifiability *I*(*m*) (bottom right) subtracting the correlation with other teams from the scale-consistency *C*(*m*). The procedure is repeated for partitions of the pitch of different sizes, leading to a value of *C*(*m*) and *I*(*m*) that depends on the number of nodes *m*.

This way, we call *“scale-consistency”* *C*(*m*) to the average correlation between matrices of the same team at each scale, where *m* is the number of divisions of the pitch. Finally, the team *“average consistency”* |*C*| is computed as the average of the scale-consistency, $|C|=\langle C(m)\rangle$. Figure 2 shows a qualitative description of the process undergone. The bottom left plot shows an example of the consistency *C*(*m*) of a team at different scales. We can observe how *C*(*m*) decreases as the number of nodes increases. The reason is that a low number of nodes leads to small networks with a low number of links (with a high weight), and, as a consequence, there is less diversity in the possible network structures. We can also observe that there are different combinations of the pitch division leading to the same number of nodes, e.g., we can have $m=20$ with $p=4$ and $q=5$ or, conversely, with $p=5$ and $q=4$.

We can also quantify how different pitch-passing networks of a team are compared with other teams’ networks. With this aim, we first obtained the scale-consistency *C*(*m*) of a given team following the process explained above. Next, we calculated the correlation *R*(*m*) of all passing networks of a team (at all scales) with the rest of the league’s teams. This can be done by considering matrices $\mathbf{A}(m)$ of Eq. (1) belonging to a given team and matrices $\mathbf{B}(m)$ corresponding to the rest of the teams. We called this parameter the *“rival correlation”* *R*(*m*), where *m* indicated the number of partitions (nodes) of the scale at which the correlation is calculated. Finally, we define the “*scale-identifiability*” *I*(*m*) of a team as the team’s scale-consistency *C*(*m*) minus the rival correlation *R*(*m*), i.e. $I({m})=C(m)-R(m)$. Finally, we can calculate the team average identifiability |*I*| as the mean value of the scale-identifiability, $|I|=\langle\sum I(m)\rangle$. Note the implications of the identifiability parameter |*I*|: Teams with a high value are those whose pitch passing networks are consistent, i.e., have a similar organization for all matches but, at the same time, are different from the rest of the teams. Again, Fig. 2 shows an example of the scale-identifiability (bottom-right plot) of a team. In this case, the function shows a maximum for a particular scale, which corresponds to a number of nodes around $m=50$. This fact reveals that pitch partitions around this scale better capture the characteristic features of this particular team’s passing networks.

## Results

**Figure 3:**
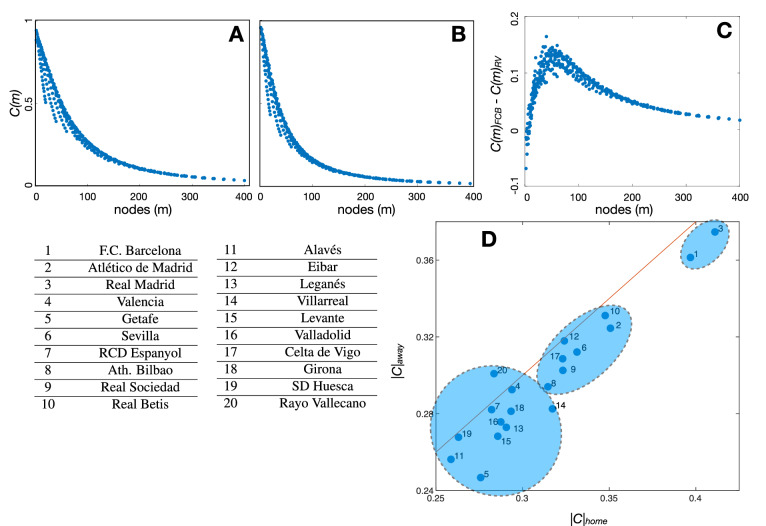
Scale-consistency *C*(*m*) of a football team, where *m* is the number of divisions of the pitch. Plots (**A**) and (**B**) show the consistency of F.C. Barcelona and Rayo Vallecano, the first and the last teams of the ranking at the end of the season, respectively. In both plots, the horizontal axis corresponds to the number of nodes *m* (areas of the pitch) at which the consistency is calculated. As we can observe, consistency decreases as the number of nodes increases. In plot (**C**), we plot the difference between the consistency of F.C. Barcelona and Rayo Vallecano as a function of the number of nodes. Differences maximize around $m \sim 50$. In (**D**), we compared the average consistency when playing at home $|C|_{home}$ or away $|C|_{away}$. The blue shadowed areas represent the three clusters detected by the k-means algorithm. Teams are numbered according to their ranking at the end of the season.

First, we computed the scale-consistency *C*(*m*) and the average consistency |*C*| of the 20 teams of *LaLiga* 2018/2019 season. Figure 3A,B shows, respectively, the scale-consistency of the teams that ranked first, F.C. Barcelona (FCB), and last, Rayo Vallecano (RV), at the end of the season. In both cases, we obtained a monotonically decaying function indicating that the higher number of divisions of the pitch, the lower the consistency of both teams. The same pattern is also reported for all teams of the competition (see Supplementary Information). However, scale-consistency has differences between teams, as we can observe in Fig. 3C, where we plot the difference between the scale-consistencies of both teams $C_{FCB}(m)-C_{RV}(m)$. As we can see, scale-consistency is higher at F.C. Barcelona, no matter what the number of divisions of the pitch is and, furthermore, the difference is maximum around $m \sim 50$, revealing that there is a particular scale where F.C. Barcelona was much more consistent than Rayo Vallecano.

Figure 3D shows the average consistency of all teams for matches played at home (x-axis) and away (y-axis). First, we restricted the calculation of the scale-consistency *C*(*m*) to matches played just at home or just away, leading to $C(m)_{home}$ and $C(m)_{away}$, respectively. Next, we obtained $|C|_{home}$ and $|C|_{away}$ by averaging both scale-consistencies. The red solid line of Fig. 3D corresponds to $|C|_{home} = |C|_{away}$ and it is plot to help the reader detecting whether a team has more consistency playing at home (points below the line) or away (points above the line). We can observe how all teams but one (Rayo Vallecano) have higher average consistencies when playing at home, since they are placed below the line $|C|_{home}=|C|_{away}$. Thus, the increase in consistency could be one more of the benefits of being the home team. Interestingly, Real Madrid and F.C. Barcelona are the teams with the highest average consistency both at home and away. These two teams achieved the third and first positions, respectively, and the highest values of consistency indicate that they are the teams whose passing networks have a more regular structure. Using the *k-means* algorithm^23^ we categorized teams into three groups, which are indicated by the shadowed regions in Fig. 3D. The three detected clusters correspond to high (1), intermediate (2), and low consistency (3). Real Madrid and F.C. Barcelona belong to cluster 1, the one corresponding to the highest consistency. Seven teams, including Athlético de Madrid (second at the end of the season), belong to cluster 2. Finally, eleven teams are assigned to the cluster with the lowest consistency (3). Interestingly, the three teams relegated to the second division (Girona, Huesca, and Rayo Vallecano) belong to this cluster.

**Figure 4:**
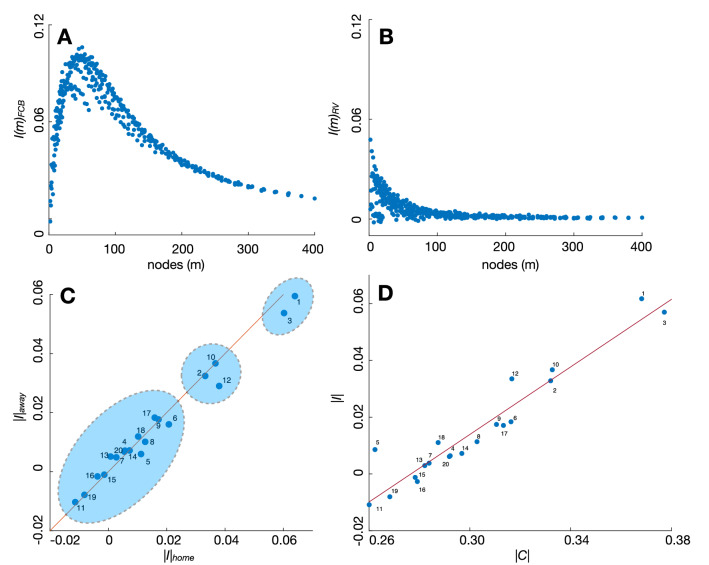
Identifiability of *LaLiga* teams. In (**A**) and (**B**), we plot the scale-identifiability *I*(*m*) of F.C. Barcelona (**A**) and Rayo Vallecano (**B**), the first and last teams of the ranking, respectively. In (**C**), we plot the average home identifiability $|I|_{home}$ vs. the away identifiability $|I|_{away}$. The solid line corresponds to $|C|_{home}=|C|_{away}$, indicating the frontier between having higher identifiability at home (below the line) or away (above the line). The shadowed areas correspond to a cluster classification given by the k-means algorithm. In (**D**), we plot the average consistency |*C*| vs. the average identifiability |*I*| of each team. In this case, the solid line is the linear regression, which has a correlation coefficient of $r=0.956$. Team numbers are the ranking at the end of the season (see Fig. 3 or Table 1 for the teams’ names).

Second, we calculated the identifiability of all teams as explained in the “Methods” section. Figure 4A,B show the scale-identifiability *I*(*m*) of, again, F.C. Barcelona and Rayo Vallecano (see Supplementary Information for the rest of the teams). In this case, we observe a different scaling behavior when the two teams are compared. F.C. Barcelona reaches high values of identifiability (as we will see, it is the team with the highest average identifiability), particularly around a pitch partition with $m \sim 50$ nodes. Therefore, it is at this scale where F.C. Barcelona passing networks are more distinguishable from the rest of the teams. Concerning Rayo Vallecano, its identifiability rapidly decays to zero, indicating that the organization of its passing networks does not have remarkable differences with the rest of the teams. Concerning the differences between playing at home or away, we observe similarities and differences from the patterns reported for the average consistency (see Fig. 4C). As in the previous case, the k-means the algorithm detects F.C. Barcelona and Real Madrid to be in the cluster of teams with the highest identifiability. However, while Real Madrid was the team with the highest consistency, the team with the highest identifiability is F.C. Barcelona. The second cluster, the one with moderate identifiability, contains only three teams: Atlético de Madrid, Real Sociedad, and Eibar. Finally, the third cluster includes the rest of the teams. The solid line in Fig. 4C corresponds to $|I|_{home}=|I|_{away}$, and we can observe how there are teams at both sides of the line, a fact not reported when carrying a similar analysis about the team’s consistency. Therefore, there is no evidence that playing at home increases the team’s identifiability. However, the two teams with the highest identifiability have better values when playing at home.

Finally, Fig. 4D shows the relation between consistency and identifiability. We plot the average consistency |*C*| of each team vs. its average identifiability |*I*| and calculate the linear regression between both variables. The high value of the correlation coefficient ($r=0.956$) confirms that teams with a high consistency also have high identifiability, despite some teams deviate from the general trend. This is the case, for example, of Getafe (ranked $\#5$), which has much higher identifiability than the one expected by its consistency.

**Figure 5:**
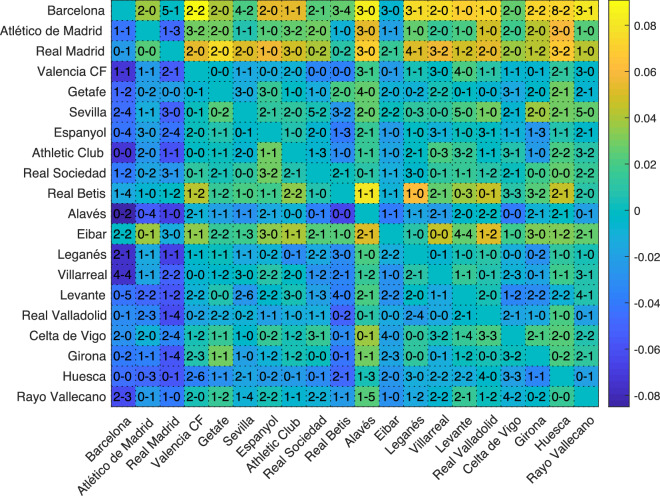
Match-identifiability for the season 2018/2019 of *LaLiga*. Teams are ordered according to their ranking at the end of the season. Rows correspond to home teams, while columns are the away teams. Each cell contains the final score of the match.

Identifiability has practical implications that may be used by coaching teams to prepare and analyze matches. For example, we can calculate our next rival’s identifiability, check if it is higher or lower when playing at home or away, and prepare the match accordingly. An opposing team with high identifiability is prone to impose its passing pattern, while a team with low identifiability will probably adapt to the opponent. Besides, we can obtain a value of the identifiability of two teams for a particular match. For example, suppose F.C. Barcelona and Rayo Vallecano have played between them. We can obtain the multi-scale passing networks of both teams for this specific match. Next, we can compute how similar are the networks of this match to the rest of the matches played by each team and obtain the match-identifiability parameter of both teams, $I^{FCB}_{match}$ and $I^{RV}_{match}$, respectively. Finally, we can calculate the difference between the match-identifiability of both teams and obtain $I_{diff}=I^{FCB}_{match}- I^{RV}_{match}$. Note that $I_{diff}$ indicates which of the two teams played more similarly to its style (maintaining its passing patterns) in that match. We computed $I_{diff}$ for all matches of the season and summarized the results in Fig. 5. Rows contain home teams, while columns correspond to away teams. Teams have been ordered, from top to bottom, according to the final ranking at the end of the season, to show the connection between identifiability and the performance of a team. Matches where the home teams imposed their passing patterns (i.e., home teams had higher identifiability) are plot in yellow, while bluish cells correspond to away teams imposing their passing patterns. In this way, we can observe how yellowish cells are mainly placed above the diagonal of the matrix, indicating that when two teams played, the one ranked in a higher position had a higher probability of imposing its playing style. If we pay attention to particular teams, we can observe that F.C. Barcelona was the team that won the “identifiability contest” in more matches, both at home and away, just followed by Real Madrid. However, it is worth mentioning that $I_{diff}$ is not always an indicator of the result of a match since there are some matches where the team that lost the match had higher identifiability. In other words, playing your way does not guarantee the victory. With this regard, we analyzed the results of a match according to the match-identifiability $I_{diff}$ and we obtained that, for the season 2018/2019, the team with highest $I_{diff}$ won in the $44\%$ of the matches, while the three points went to the team with the lowest $I_{diff}$ in the $27\%$ of the cases.

## Discussion

We have introduced a new methodology to quantify the consistency and identifiability of football teams. Our approach focuses on the organization of pitch passing networks, whose nodes are different areas of the pitch connected through the completed passes between any two regions. Pitch passing networks contain information about how teams move the ball along the match and what are the most connected areas of the pitch. Furthermore, they contain spatial information that is lost when the nodes of the passing networks are the players themselves^14,18^. It is worth mentioning that there is no unique pitch passing network for a given match, but a “family” of them. The reason is that the pitch can be divided into partitions of different sizes, leading to different pitch passing networks, with a different number of nodes and, consequently, with a different structure. Within this framework, we defined the team’s consistency as the ability to maintain the structure of pitch passing networks from match to match. Consistency was quantified by, first, obtaining the adjacency matrices associated with pitch passing networks of a given team and, second, calculating the cross-correlation of these matrices. We repeated this process with partitions of the pitch of different sizes, from $m=4$ areas to $m=400$. In this way, we obtained the scale-consistency *C*(*m*) of football teams, indicating how similar pitch passing networks are at different scales. The team consistency |*C*| obtained as the average of the scale-consistency allows to rank teams according to this parameter and, furthermore, to distinguish between the behavior of the team in matches played at home or away. The analysis of Spanish football teams paying at LaLiga during season 2018/2019 revealed that Real Madrid and F.C. Barcelona were the most consistent teams, while Alavés and Getafe were the most inconsistent. It is worth mentioning that consistency (or identifiability) is not a proxy of team’s performance, but a quantifier of how a team maintains a particular passing pattern. Therefore, the value reported for each team requires a particular interpretation. On that sense, Getafe is a team with a playing style characterized by an intense pressure at higher positions of the field, adapting to the specific formation of their rivals and, as a consequence, having a low consistency (and identifiability). However, Getafe qualified fifth at the end of the season, a commendable position according to its budget. Concerning the location of the match, all teams played more consistently at home than away, except Rayo Vallecano, the team that finished the championship at the last position. These results indicate that home teams are prone to maintain a playing pattern compared to away teams.

Beyond consistency, we investigated how the organization of pitch passing networks was related to each particular team. Our main aim was not to categorize playing styles but to quantify how different are the passing patterns of a given team from the rest of its rivals. With this target, we computed the identifiability of football teams by, first, quantifying their consistency and, second, subtracting the similarity with the pitch passing networks of the rest of the teams. Teams with high identifiability fulfill two requirements: (1) they have consistent passing networks during the season and (2) their passing networks are different from the rest of the teams. Again F.C. Barcelona and Real Madrid were the two teams with the highest identifiability. However, the Catalan team led the identifiability ranking, instead of Real Madrid, which had the highest consistency. Furthermore, we found that specific teams had an optimal partition of the pitch (around $m \sim 50$ nodes) at which the team’s identifiability was higher. This optimal scale was reported at the identifiability of F.C. Barcelona, Real Madrid, Atlético de Madrid and Real Betis, all of them having high values of the identifiability parameter. The fact that an optimal scale exists has significant consequences on the definition of pith networks: Divisions of the pitch leading to a too high or too low number of nodes may lose information about the passing patterns. Interestingly, we did not find a standard behavior when comparing the identifiabilities of home/away teams, i.e., some teams are more identifiable at home but others away. The comparison between consistency and identifiability showed a positive correlation, despite some teams, such as Getafe, deviated from the general trend. The interpretation of such deviations should be carried out by football professionals/experts with knowledge about each team’s specificities and the circumstances surrounding their performance (e.g., a change of the coaching team or player injuries). Finally, we showed how it is possible to obtain an identifiability value of a team for each match, which can be used to assess which team imposed its passing pattern during the game. This information could also help to prepare the next match according to the particular identifiability of the rival. The match-by-match analysis showed that F.C. Barcelona was the team imposing their passing patterns more frequently both at home and away, followed by Real Madrid. However, having higher identifiability than the rival at a given match was not necessarily an indicator of a victory. Although teams placed at a more top position of the ranking generally had higher match identifiability than their rivals, we observed some matches where the final score did not correlate with this difference.

It is worth also mentioning the limitations of our methodology. On the one hand, we only used the information contained in the passes made by a team. This fact unavoidably limits the detection of the particular signature of teams, since there are a diversity of other actions contributing to a team’s playing style. Indeed, there is space for improving the identification of playing styles once the analysis goes beyond the limitations of classical team formations, such as 4-4-2 or 4-3-3. Therefore, further improvements in our methodology should consider including information about other actions such as shots, tackles, or dribbles. Another shortcoming of the consistency and identifiability parameters is that we need a certain number of matches to quantify them. Therefore, although we could obtain both parameters before the end of the season, they can not be calculated at the beginning of the championship since we would not have enough matches to define each team’s average consistency and identifiability. Furthermore, there are unavoidable changes in the team structure that may affect the team’s passing pattern. For example, a given team may change its coach in the middle of the season and have low identifiability values due to a change in its playing style. With this regard, it is always necessary to know each team’s context to interpret team consistency or identifiability adequately.

Finally, we believe that further studies could develop in more detail the information contained in both the identifiability and consistency parameters. For example, it would be possible to evaluate what regions of the pitch are contributing the most to these parameters. In this way, coaching teams could benefit from the detection of the areas of the pitch where our team (or the rival) is playing as expected and others, where the behavior deviates from the plan. On the other hand, it would be possible to obtain a real-time estimation of the identifiability parameter during a match and analyze at what moments a team (or its opponent) is playing as expected, being a valuable information for making decisions and/or find explanations in the subsequent analysis. Hopefully, in the years to come, we will see more sophisticated definitions of playing styles, not only in football but in other team sports where the way players interact crucially influences team performance.

## Supplementary information

Supplementary information.
