# Supplementary material for: Consistency and identifiability of football teams: a network science perspective
Source: Sci Rep. 2020 Nov 12;10:19735. doi: 10.1038/s41598-020-76835-3 (PMC7661721; doi:10.1038/s41598-020-76835-3)
Supplement: Supplementary file 1 — Supplementary information. [file 41598_2020_76835_MOESM1_ESM.pdf]

Supplementary Information:

Consistency and identifiability of football teams: A network science perspective, by D. Garrido, D. Ruiz Antequera, J. Busquets, R. López del Campo, R. Resta Serra, S. Jos Vielcazat, and J.M. Buldú

Contents

S1 Scale-consistency $C(m)$ of all teams of *LaLiga* during the season 2018/2019 **2**

S2 Scale-identifiability $I(m)$ of all teams of *LaLiga* during the season 2018/2019 **3**

S1 Scale-consistency $C(m)$ of all teams of *LaLiga* during the season 2018/2019

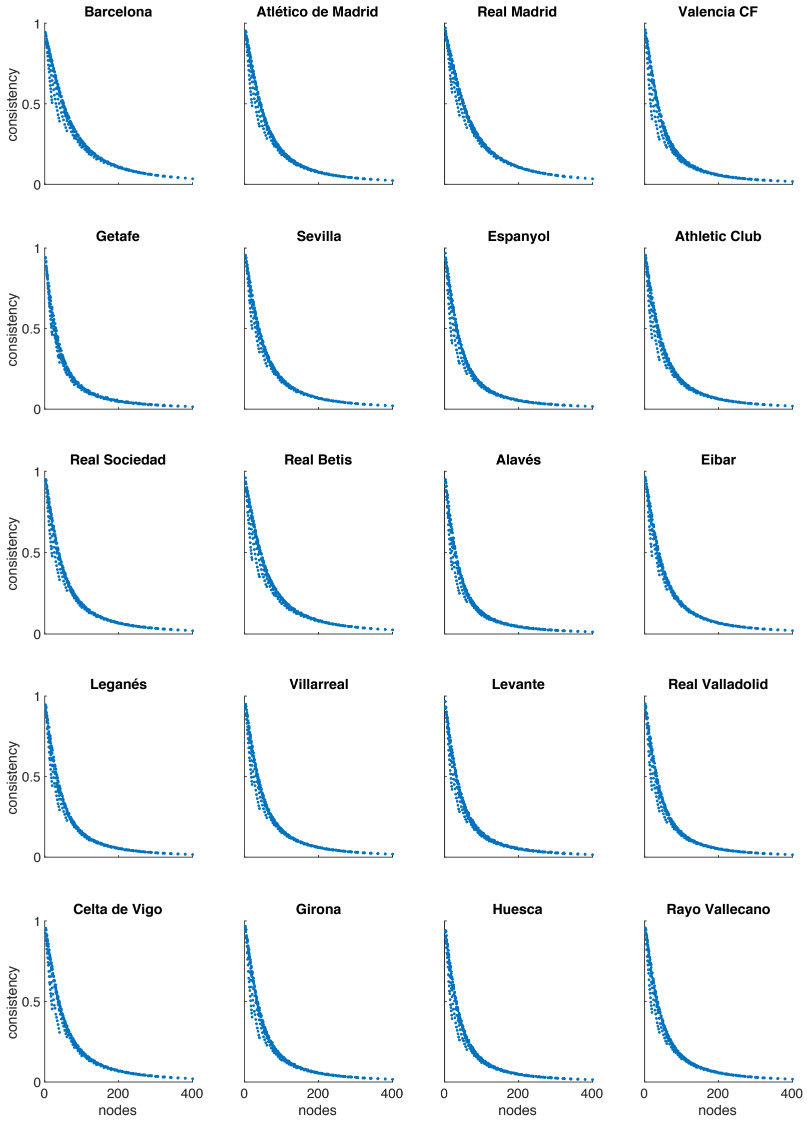

Figure S1. For each team, scale-consistency $C(m)$ as a function of the number of nodes m of the pitch network. In all cases, we observe monotonically decaying functions, despite the decay rate is not equal for all teams, as shown in the example of Fig. 3C of the main text.

S2 Scale-identifiability $I(m)$ of all teams of *LaLiga* during the season 2018/2019

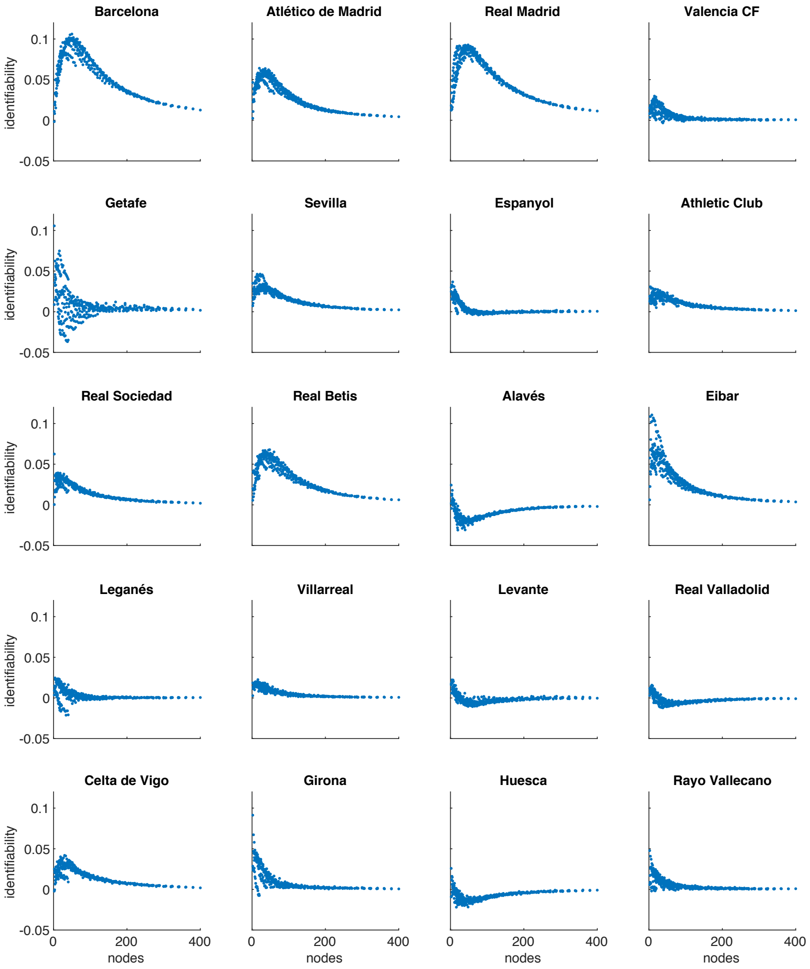

Figure S2. For each team, scale-identifiability $I(m)$ as a function of the number of nodes m of the pitch network. As we can observe, teams with the highest identifiability are prone to have a maximum value around partitions with $m \sim 50$ nodes. On the contrary, the majority of teams with low identifiability have values close to zero, no matter the number of divisions of the pitch.
